# Role of Stone Heterogeneity Index in Determining Success of Shock Wave Lithotripsy in Urinary Calculi

**Published:** 2021-03-24

**Authors:** Nadeem Iqbal, Aisha Hasan, Ahsan Nazar, Sajid Iqbal, Mohammad Haroon Hassan, Behzad Saeed Gill, Rabiyya Khan, Saeed Akhter, Rodrigo Suarez-Ibarrola

**Affiliations:** ^1^Department of Urology and Kidney Transplant, Pakistan Kidney Institute, Shifa International Hospital, Islamabad, Pakistan; ^2^Riphah International University, Rawalpindi, Pakistan; ^3^Department of Rehabilitation, Pakistan Navy PNS Hospital, Karachi, Pakistan; ^4^Department of Urology, Faculty of Medicine, University of Freiburg, Germany

**Keywords:** shock wave lithotripsy, computed tomography scan, stone fragility, stone heterogeneity index, urinary calculi

## Abstract

**Introduction:**

Various stone factors can affect the outcome of shock wave lithotripsy (SWL). A novel factor called the stone heterogeneity index (SHI) may have an impact on stone free rates. The objective of this study was to assess the role of SHI in SWL outcomes.

**Methods:**

Patients’ medical records were reviewed for the collection of data variables. They were subjected to SWL, using an electromagnetic lithotripter machine (Storz Modulith SLX-MX). Computation of mean stone density (mean value of the Hounsfield units) and SHI was accomplished by generating elliptical regions of interest on the computed tomography (CT) scan images. Grouping was performed on the basis of stone free and failure outcomes. Relevant statistical tests were applied for continuous and categorical variables. P ≤ 0.05 was considered statistically significant.

**Results:**

Overall, 385 subjects were included having a mean age of 38.4 ± 14.7 years. The cohort comprised 276 (71.7%) males and 109 (28.3%) female patients. A total of 234 (60.8%) patients were rendered successful (stone free after one session) while 151 (39.2%) of the patients were declared to have failed the SWL procedure. Stone length, stone density, and SHI values were 13.7 ± 7.6 mm, 935 ± 404, and 201 ± 107, respectively. The stone density, SHI, and stone length were significantly different between the two groups (p-values of 0.001, 0.02, and 0.04, respectively).

**Conclusions:**

SHI can be a helpful CT scan-based parameter to assess stone fragility. It can help clinicians in the judicious selection of patients before implementing SWL procedure.

**Relevance for patients:**

Non-contrast CT-based stone parameters have been found to be effective for predictions of outcomes. SHI can be a helping tool to better predict SWL success rates when treating the renal stones.

## 1. Introduction

Extracorporeal shock wave lithotripsy (SWL) is an essential component in the urologist’s armamentarium for managing renal stones. In recent years, it has gained increasing popularity due to its minimal invasiveness [1,2]. Furthermore, it can be carried out under sedation as an out-patient procedure and is considered a straightforward procedure to perform with overall few complications [3,4]. Despite these advantages, it is vital to note that certain challenges require further investigation [5,6]. One of the main challenges includes the variance of SWL outcomes reported in the literature [7]. This variation in efficacy might be attributed to technical issues, criteria variations for reporting the success of SWL, and substandard criteria adopted for patient selection. This has also raised concerns about the modality’s cost-effectiveness.

The European Association of Urology guidelines recommend SWL to be considered among the first-line options for the treatment of renal stones <2 cm. However, various factors have been identified to negatively influence the efficacy of SWL, for instance, lower pole stones, staghorn stones, and multiple calculi. Considering these limitations, researchers are in the quest of predictive factors that might improve patient selection and, therefore, SWL outcomes. Recently, computed tomography (CT)-based factors such as a skin-to-stone distance (SSD) ≥10 cm and stone Hounsfield units (HU) >1000 have been implicated in adverse SWL outcomes [6,7].

Due to these newly found factors, some calculi are either partially or completely resistant to SWL, entailing ancillary procedures that result in additional costs [1,4,8]. The mean stone density (MSD) has emerged as a vital and independent predictive factor of SWL net results [8,9]. MSD (mean value of the HU) is measured on a non-contrast CT (NCCT) using a picture archiving and communication system (PACS) in a specific stone area [10]. In general, such a picture archiving mechanism can furnish additional pixel statistical measurements such as the maximum, minimum, and standard deviation values of HU. Statistically speaking, the standard deviation quantifies the proportion of variation or distribution of given data values, so an excessive standard deviation stipulates the data points to be scattered over a wider range of values. Similarly, a high value of standard deviation for HUs might advocate heterogeneity in the composition of calculi. Lee *et al*. stipulated that despite similar MSD there might be composition variations among different calculi [11]. They assumed a heterogeneous stone to be more fragile as compared to a homogeneous stone. As a result, they proposed and developed the stone heterogeneity index (SHI). The SHI was designated as the standard deviation of MSD when measured on NCCT. In this study, we adopted their novel concept into our clinical practice for the treatment of renal and upper ureteral stones.

## 2. Methods

### 2.1. Patients

We analyzed data from a single-center prospective cohort that underwent SWL from June 2016 to June 2019 at the Department of Urology of the Shifa International Hospital in Islamabad, Pakistan. The study was approved by the local ethical committee before its commencement. Demographic and clinical data variables, such as patient age, gender, stone laterality, stone location, and body mass index (BMI), were extracted by reviewing patients’ electronic medical records.

All patients were radiologically diagnosed after obtaining their full medical history and being physically examined before undergoing SWL. Radiological assessment was accomplished with kidneys, ureter, and bladder (X-ray KUB) radiography and NCCT. Urine culture was obtained before the procedure to confirm that patients’ urine was sterile. Other biochemical parameters included blood biochemistry, complete blood count, and coagulation parameters before the procedure in all patients. Furthermore, patients’ informed consent was acquired before SWL treatment.

Inclusion criteria constituted stone size 5 mm–20 mm, radiopaque calculi on plain radiography, and ureteric or renal location. Only those patients were included who had NCCT available for review and the rest were excluded from the study. Exclusion criteria consisted of patients of <18 years of age, subjects suffering from active urinary tract infection, presence of anatomical renal abnormalities, multiple renal calculi, renal inadequacy, and patients having solitary kidney and an antecedent SWL procedure or ipsilateral renal stone surgery. A total of 385 patients were incorporated in this study based on the aforementioned criteria.

### 2.2. SWL technique

The patients were subjected to SWL, using an electromagnetic lithotripter (3^rd^ generation; Storz Modulith SLX-MX). Patients were placed under supine position. Fluoroscopy was used to determine the location of stones which was assisted by ultrasonography (model Aloka SSD-Thousand; 1000). The frequency of the shock waves delivered was set at 90 shocks waves per minute. Initially, 500 shocks were delivered at the energy level 2 to achieve vasoconstriction and then a gradual ramping up of these waves was set to an energy level of 3 and 4 for next 2000-2500 shocks. We labeled patients being stone-free if their plain X-ray (KUB) or ultrasound KUB obtained after 3 months of the last lithotripsy session revealed complete absence of stone fragments or if there were only clinically insignificant residual stone fragments measuring ≤4 mm.

### 2.3. Stone features on CT (non-contrast)

**Figure 1:**
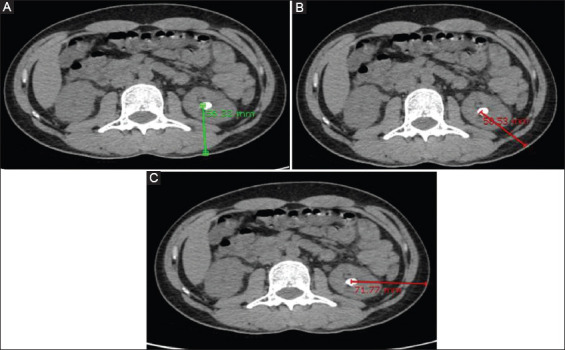
(A) The computation of skin-to-stone distance at 90° on an axial scan of non-contrast computed tomography. SSD: Skin-to-stone distance. (B): The computation of skin-to-stone distance at 45° on an axial scan of non-contrast computed tomography. SSD: Skin to stone distance. (C): The computation of skin-to-stone distance at 0° on an axial scan of non-contrast computed tomography. SSD: Skin-to-stone distance. Average of skin to stone distance measured at three angles (0°, 45, and 90°) was considered as the skin to stone distance.

**Figure 2:**
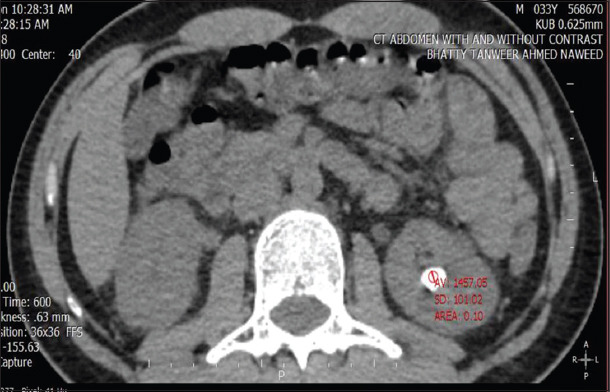
Mean stone density and standard deviation of stone density (also called stone heterogeneity index) in red elliptical area on axial view of computed tomography scan.

Stone attributes included the size, stone location, SSD, MSD, and SHI. We utilized a multi-detector CT scanner (3.0 mm/120 kV/200 mAs, Aquilion One, Merge Health Care 2006 & 2010, Chicago, IL, USA) for imaging and subsequent measurements. Stone size was computed as the stones’ largest diameter (on the axial/coronal plane of NCCT), and for assessing the SSD we followed the methodology portrayed by Pareek *et al*. (Figure 1A -C). Computation of the MSD was accomplished on an axial CT image by generating an elliptical region of interest in the stone area on the CT scan, portraying the stone in its longest dimension. MSD was described as the mean value of Hounsfield’s units in the region of interest, while the SHI was designated as the standard deviation of Hounsfield’s units in the same specified region of interest (Figure 2). Special attention was given not to include any soft tissue while measuring the stone density [12].

### 2.4. Procedural outcomes and operational definitions

A successful lithotripsy procedure was considered when patients attained stone-free status or harbored clinically trivial and unimportant residual fragments (size ≤4 mm when computed in the longest diameter) after 3 months of follow-up after the first session without the necessity for ancillary interventions. Follow-up was done with plain KUB radiography and ultrasonography.

### 2.5. Statistical analysis

Following data collection, the Statistical Package for the Social Sciences, version 16 was used for data analysis (SPSS Inc.; Chicago, IL, USA). Continuous variables such as calculus size, subjects’ age, and stone density were compared with the help of a Student’s t-test and Mann–Whitney test where applicable**.** Categorical values of variables were assessed by applying Pearson’s Chi-squared test. *P* < 0.05 (Two-tailed) was gauged to be statistically significant while making these comparisons.

## 3. Results

Overall, 385 subjects were included in this study who had the post procedural follow-up and the CT-based stone parameters were available in the records. Their mean age was 38.4 ± 14.7 years. There were 276 (71.7%) male and 109 (28.3%) female patients. A total of 234 (60.8%) patients were rendered successful (stone-free after one session) while 151 (39.2%) patients were declared to have failed the SWL procedure. Left side stones were encountered in 200 (51.9%) and right-sided in 185 (48.1%) patients. Location of stones were 41 in upper pole, 68 in mid pole, 141 in lower pole, 85 in the renal pelvis, 49 in upper ureter, and 1 in mid-ureter.

Mean stone length was 13.7 ± 7.6 mm. MSD was 935±404. Mean SHI was 201 ± 107. Mean SSD was 9.5 ± 2.4 cm. No differences were observed regarding age, gender, and stone laterality across the two groups (Table 1). Similarly, it is evident from Table 1 that there was no notable difference across the successful and failure groups regarding the SSD. However, the SHI and stone length were significantly different between the two groups (Table 1).

**Table 1:** Demographic characteristics of patients

Parameters	Unsuccessful group	Successful group	p-value
Age	38.37±14.1	38.43±15.15	0.9
Gender (n)			
Male (n)	110	166	0.6
Female (n)	41	68	
Stone length (mm) Mean±SD	14.72±7.09	13.03±8.5	0.04
SSD (cm) Mean±SD	9.6±2.3	9.4±2.5	0.7
Stone density (HU) Mean±SD	1057.2±381.5	857.1±399.3	0.001
SHI (HU)	187.7±121.4	217.1±101	0.02
Stone location, n (%)			0.5
Upper pole	13	28	
Mid pole	28	40	
Lower pole	51	90	
Pelvis	36	49	
Upper Ureter	23	26	
Stone laterality, n (%)			0.17
Right (n)	79	106	
Left (n)	72	128	

HU: Hounsfield units; SD: Standard deviation; SHI: Stone heterogeneity index; SSD: Stone-to-skin distance

On subdivision of the stone size categories (Table 2), it was found that in subjects with a stone size in the range of 10–15 mm and 15–20 mm the stone-free rate for one session were significantly affected by the difference in SHI (one-session success rate was 59.86% in stone category 10–15 mm and 51.57% in stone category 15–20 mm size). However, one-session success rate was not affected significantly by the SHI in the stone size category of 5-10 mm (Table 2).

**Table 2:** SHI and its effect on success rate in different stone size groups

Stone size category	No patients stone free	No patients Stone failure	SHI stone free group	SHI stone failure group	p- value
5–10 mm	94	44	225.2 ± 104.7	218.7 ± 138.7	0.7
10–15 mm	91	61	210.8 ± 90.1	174.1 ± 118.1	0.03
15–20 mm	49	46	206.9 ± 105.4	157.8 ± 85.2	0.01

SHI: Stone heterogeneity index

When sub-analysis was performed based on different stone density categories, it was observed that single-session success rates were 73.23% in patients having stone density up to 500 HU (Table 3). However, the SHI was not notably different between stone-free and failure subjects for the stone density category up to 500 HU (Table 3). In contrast, it was clearly seen that there was a sharp difference between SHIs for stone-free and stone failure patients in the subgroup of patients having a MSD of 500–1000 HU (Table 3). Moreover, the SHI was significantly different across stone failure and success patients, even in the higher stone density subgroup (1000–1500 HU), *P* = 0.02 (Table 3).

**Table 3:** SHI and its effect on the success rate in different stone density groups

Stone density category	No patients stone free	No patients stone failure	SHI stone free group	SHI stone failure group	p- value
Below 500 HU	52	19	164.6±78.9	121.9±101.2	0.06
500–1000 HU	96	48	222.03±91.1	172.4±88.06	0.002
1000–1500 HU	86	84	227.2±95.3	194.5±92.7	0.02

SHI: Stone heterogeneity index

**Figure 3:**
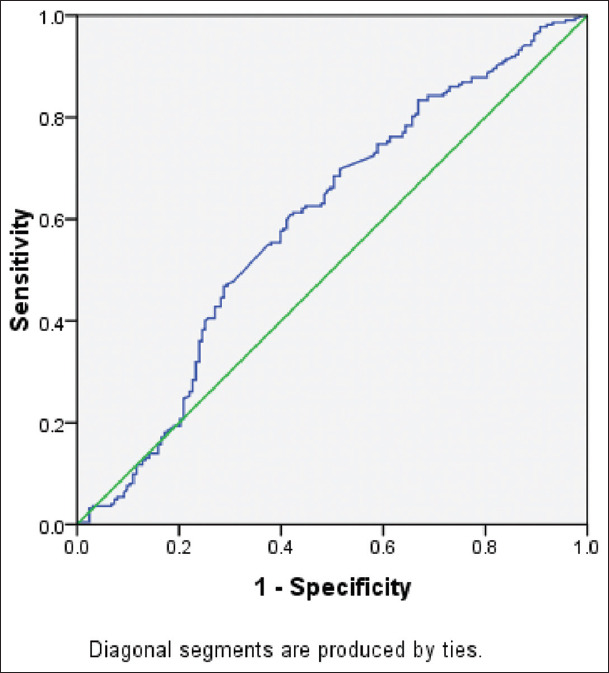
Receiver operating characteristic of stone heterogeneity index for shock wave lithotripsy success

After generating receiver operating characteristic curves, a cutoff value for SHI was found to be 213. It had a sensitivity of 0.67 and a specificity of 0.60. The area under the curve was 0.60 (Confidence Interval 0.531–0.673), which demonstrated greater sensitivity and specificity values (Figure 3).

## 4. Discussion

In the present study, we investigated the concept of stone radiologic features seen on CT scan images, the heterogeneity and found important observations. To the best of our knowledge, this is the first study where the confounding factor of SSD was abolished between the stone-free and stone failure groups due to the patients included. In spite of that, looking at Table 1, it can be observed that the overall stone size was significantly different between stone-free and stone failure groups (Table 1). Likewise, stone density and stone heterogeneity indices were also notably different across stone-free and failure groups (Table 1).

It has been observed over the years that larger stone size (especially >10 mm) or a higher stone density (especially more than >1000 HU) have been considered factors that promote resistance to SWL. Similarly, the stone size appears to be the most dominant factor in foretelling SWL success [13,14]. Our results showed that stone size was notably different across stone-free and failure groups (*P* = 0.04, Table 1) that is in congruence with preceding studies. Some of the recent developments in the quest for new stone related factors have pointed toward certain features like higher SHI that can help clinicians in understanding more about formulating our decisions in favor of achieving maximum success rates for stone treatment. Thus, promising results can be obtained despite so-called unfavorable stone features (higher stone size or density).

Recent studies suggest that the SHI can play a vital role as a parameter for computing the extent of stone fragility. Thus, it could be relied on to complement clinical decisions based on some already known predictive variables such as stone size and stone density. As mentioned earlier, SHI (heterogeneity index of stone) can be effortlessly measured by making use of PACS. CT scan-based parameter stone density has been studied and used extensively in the past few years as a useful tool to portray a general idea about the hardness of a particular stone [15]. However, there are some reservations being raised recently by some researchers that the mean density for a particular stone is merely an average value and as such, it cannot represent the heterogeneousness due to particular stone composition**.** On the other hand, the term standard deviation is the square root of its variance. The SHI radiologically portrays the heterogeneity of the stone (the standard deviation computed for HU within a particular region of interest). As such, the SHI can give a picture of the intrinsic diversity within the stone, depicting the variation in the stone’s composition as well as the inner architectural variations (morphological heterogeneity). In a study by Jing *et al.*, it was found that only 37.4% of renal stones were pure while the majority depicted a mixed (62.6%) composition having calcium oxalate as the most frequent component [16]. It is important to know that in some instances despite the homogenous composition of stone minerals the type of the inner architecture of stones may affect SHI values [17]. These may be seen externally as variations in shape or contour irregularities (smooth, round, speculated, or mulberry stones). Oftentimes, the space inside the stone might be filled with air/water and could affect SHI values [18].

In recent years, various studies portrayed that the composition of renal stones might be determined by utilizing the concept of stone density; however, this might not be enough to allow a reasonable projection of the stone’s fragility. This might be attributed to disparities in the internal structural arrangement as well as the overall stone’s chemical composition [19]. Formerly, various studies threw light on CT scan-based variables (parameters) and their possible relation to SWL net results. These factors include mainly stone volume, SSD and stone density (HU) from CT scan imaging assessments. The MSD has been of much interest in terms of its possible role as a distinctive predictive tool to foretell SWL outcomes. Several recent studies manifested MSD to have a vital association with SWL outcomes. Some of these studies implicated stones of more than 900 HU to be a cause of SWL failure [20]. Nakasato *et al*. mentioned SWL procedural success to be strikingly higher when the density was <815 [21]. Ouzaid *et al*. inferred that stones of more than 970 HU had higher chances of SWL failure [22]. El-Nahas pointed out that a MSD value of more than 1000 HU was a vital tool to predict SWL procedural failure [23]. They were of the view to offer substitute treatment modalities in such patients [23]. The role of stone size has been confirmed by various studies [23]. Apart from this, the role of SSD is controversial with varying results [24]. As far as SHI is concerned, a recent study where stones with a mean density of more than 1000 HU were analyzed, demonstrated that stone-free patients manifested remarkably higher SHIs compared to subjects who had failed SWL. In our study, the stone density and SHI were significantly different between the two groups (Table 1). We used one lithotripter for all subjects while Lee *et al*. used two unalike SWL machines that could have created bias in their observations [11].

In a study by Lee *et al.*, subjects having stone size more than 10 mm had a one-session success rate of 50.2%. They further observed that SHI values were strikingly dissimilar among subjects who had successful and failed SWL procedure (280 ± 116 HU vs. 205 ± 86 HU) [11]. In the present study, stone size categorization was done for analysis (as shown in Table 2) and it was found that in subjects with a stone size in the range of 10–15 mm and 15–20 mm the stone-free rate for one session was significantly affected by the difference in SHI (Table 2). However, one-session success rate was not affected notably by the SHI in the stone size category of 5–10 mm size (Table 2). Our results for the stone size category 10–15 mm were better compared to the Lee *et al*. study while the size category of 16–20 mm had findings similar to their results. They might have differences in results due to the use of two different SWL lithotripters, while we used the same machine for all cases [11].

According to Lee *et al.*, in subjects who had higher stone density (MSD ≥ 1000 HU), the SHI value was strikingly higher in one-session success group compared to the failure group (308 ± 92 HU vs. 251 ± 55 HU). In the present study, the sub-analysis done based on different stone density categories manifested that the SHI was not notably different between the stone-free and failure subjects for a stone density category up to 500 HU (Table 3). In contrast, it was clearly seen that there was a sharp difference between SHIs for stone-free and stone failure patients in the subgroup of patients having a MSD of 500–1000 HU (Table 3). Moreover, the SHI was significantly different across stone failure and success patients, even in the higher stone density subgroup (1000–1500 HU) (Table 3). We took into account the stone density subgroup of 500–1000 HU and the effect of SHI on outcomes. We found that even in this stone density category SHI played a vital role. Lee *et al*. did not take this into account in this subgroup. They merely described the groups in terms of less than and more than 1000 HU and the effect of SHI on outcomes of SWL. They stated that there was no role of SHI for the stone density group below 1000 HU subjects. On the contrary, when we further sub-categorized the subjects into <500 and 500-1000 HU cases it was clear from results that SHI played a role even in 500–1000 HU range stones. Xun *et al*. also suggested that the presence of lower-density portions might be vital for ESWL successful outcomes [25]. They mentioned that a considerable heterogeneity of stone density might enhance fragmentation. However, they too had a retrospective study having potential bias regarding patients’ selection. Moreover, they studied only 100 patients, which is a very small sample for making inferences regarding the subject matter.

The main limitation of the study is that it was a single-center experience. Nevertheless, this study has particular strengths as having a prospective design and subcategorizing the subjects into the different stone size and stone density groups while comparing the effects of SHI in all these subgroups. Such subgrouping has not been studied in the literature before. However, further prospective multicenter studies are required to authenticate our findings on the interrelation between SHI and SWL net results. Furthermore, the clinically pertinent cutoff value needs to be formulated in light of findings from prospective multicenter studies. This might help in the appropriate selection of subjects for SWL treatment. Finally, new avenues for research studies regarding chemical and inherent structural inspection of renal stones should be encouraged in collaboration with clinical urologists to help in devising scores or nomograms for better decision-making while treating renal stone patients.

## 5. Conclusions

It is inferred that the SHI can be a helpful CT scan-based parameter to assess stone fragility. Moreover, it will furnish additional information besides stone size and density to help the clinician in selecting the appropriate treatment procedure. Thus, it can be helpful in the selection of subjects judiciously before implementing the SWL procedure.
